# Soil microbiome composition is highly responsive to precipitation and plant composition manipulations in a field biodiversity experiment

**DOI:** 10.3389/frmbi.2025.1460319

**Published:** 2025-01-31

**Authors:** Haley M. Burrill, Susan M. Magnoli, James D. Bever

**Affiliations:** ^1^ Institute of Ecology and Evolution, University of Oregon, Eugene, OR, United States; ^2^ Kansas Biological Survey and Center for Ecological Research, University of Kansas, Lawrence, KS, United States; ^3^ Laboratoire d’Écologie Alpine, The National Center for Scientific Research (CNRS), Univ. Grenoble Alpes, Gières, France

**Keywords:** microbiome, fungi, plant soil interactions, biodiversity, climate change

## Abstract

**Introduction:**

Climate change and plant biodiversity loss have large impacts on terrestrial ecosystem function, with the soil microbiome being primary mediators of these effects. The soil microbiome is a complex system, consisting of multiple functional groups with contrasting life histories. Most studies of climate forces and plant biodiversity effects on microbiome consider the perturbations and the microbial functional groups in isolation preventing us from understanding the full picture of the relative and differential impacts of perturbations on microbial functional groups.

**Methods:**

We measured changes in multiple microbial communities with different functionality, including plant mutualists and pathogens, after three growing seasons in a full-factorial experiment manipulating precipitation (50%, 150% of ambient), plant diversity, and plant composition. Using amplicon sequencing to characterize the response of fungi, arbuscular mycorrhizal fungi, bacteria and oomycetes, and we found that composition of all microbial groups differentiated strongly between precipitation treatments.

**Results:**

Oomycete and bacterial diversity increased with 150% precipitation, while AM and saprotroph fungal diversity decreased. Microbial differentiation in response to plant family and plant species composition was stronger after the third growing season than observed after year one. However, microbial response to plant species richness was weaker in year three. Microbiome response to plant composition was largely independent of the response to precipitation, except for oomycetes, which had greater response to plant composition in high precipitation.

**Discussion:**

These findings build upon prior findings that these microbial community members differentially respond to plant community compositional treatments, by measuring the response over 3 years and with the addition of precipitation treatments. We find that both changes in climate and plant composition can drive major differences in soil microbiome composition, which can feed back on plant community structure and alter ecosystem function.

## Introduction

Global climate change has large impacts on terrestrial ecosystem function, where fluctuations in precipitation patterns can range from extreme drought to high magnitude rainfall events in ecosystems that are not adapted to these conditions. At the same time, ecosystem function is threatened by rapid biodiversity loss (Tilman et al., 2012). The possibility that climate change and biodiversity have compounding effects on ecosystem function highlights the necessity of considering both factors simultaneously. Predictions of such impacts can be improved by better understanding the potential mechanistic mediators of biodiversity and climate change on ecosystem processes. Ample research demonstrates how soil microorganisms play critical roles in ecosystem function (Austin et al., 2014; Dubey et al., 2019; Podzikowski et al., 2024) and biodiversity maintenance (Van Der Heijden et al., 2008; Bever et al., 2015) and are therefore likely candidates to mediate the joint impacts of biodiversity and climate change effects on ecosystem function. Thus, it is essential to understand how the soil microbiome, including functionally distinct microbial groups, respond to climate perturbations, along with changes in plant biodiversity and composition.

The soil microbiome has been shown to be highly responsive to changes in precipitation (Barnard et al., 2013; Engelhardt et al., 2018). The richness, abundance, and composition of bacteria and fungi, including fungal pathogens (Coulhoun, 1973; Talley et al., 2002, Delavaux et al., 2021a) and arbuscular mycorrhizal (AM) fungi (House and Bever, 2018), and oomycetes (Van West et al., 2003, Delavaux et al., 2021a), have been shown to change with precipitation. Although both bacteria and fungi are responsive to increased precipitation, fungi have been found to be more tolerant of drought conditions than bacteria (Barnard et al., 2013; Engelhardt et al., 2018). At the same time, some fungal pathogens (e.g. rust, Froelich and Snow, 1986; root rot Wyka et al., 2018; Bevacqua et al., 2023) and saprotrophs (Delavaux et al., 2021a) have been found to proliferate in wetter conditions. In addition, terrestrial oomycetes, which are often plant pathogens, have been found to increase in diversity in wetter conditions (Delavaux et al., 2021a), as might be expected from their water-dependent life cycle (Thines, 2018). Thus, These differential responses to precipitation have major implications for microbiome feedbacks on plant communities, such as increased reliance on AM fungal partners under drought conditions (Stahl and Smith, 1984; Schultz et al., 2001; Auge, 2001; Marulanda et al., 2003) and potentially greater impacts of pathogens in wetter condition. Thus, identifying the relative sensitivities of functionally and taxonomically distinct soil microbial groups to major precipitation changes is essential to understanding how microbiome-driven functions might shift with elongated drought periods and more drastic rainfall periods. No study to date has measured the breadth of microbial functional groups to experimental alteration of precipitation.

The soil microbiome is also highly responsive to plant community composition. Increased plant species richness can generate an increase in microbial diversity (Lamb et al., 2011; Burrill et al., 2023), as plant species’ microbiomes often differ depending on root architecture (Saleem et al., 2018), root exudates (Turner et al., 2013), and other functional traits such as defense mechanisms (Gilbert and Parker, 2016). In particular, plant pathogens often specialize on a plant host species to circumnavigate different host traits, exudates, and defenses (Gilbert and Parker, 2016). Both fungal pathogen and oomycete diversity have been shown to increase with plant species richness, and their community composition has been shown to differentiate between plant families, both suggesting host-specificity (Burrill et al., 2023). Additionally, similar phylogenetic signals to those of plant pathogens have been detected in saprotrophs (Malik et al., 2022; Kaplan et al., 2020), and may be expected for AM fungi due to host-specific differentiation (Mangan et al., 2010a; Bever, 2002). Furthermore, studies demonstrate support for saprotrophic microbe mediation of the positive relationship between decomposition rates and plant species richness (Glassman et al., 2018; Podzikowski et al., 2024). Thus, the functional breadth of the soil microbiome can be sensitive to plant community composition.

The relative sensitivities of taxonomic and functionally distinct groups of soil microorganisms to plant communities are particularly important because they may have implications for feedbacks on plant fitness, composition, and productivity (Bever et al., 2012; Crawford et al., 2019; Wang et al., 2019). For example, specialized pathogens can accumulate in plant communities with low diversity (e.g. monoculture), driving negative plant-soil feedbacks (Mills and Bever, 1998; Bauer et al., 2015), potentially mediating plant species coexistence (Bever et al., 1997) and productivity benefits of plant diversity (Wang et al., 2023; Collins et al. 2020). These feedbacks have been shown to be strongest when plant phylogenetic distance increases (Crawford et al., 2019), consistent with phylogenetic signaling of pathogen specialization on plant hosts (Parker et al., 2015; Gilbert and Webb, 2007; Gilbert and Parker, 2016). Additionally, changes in microbiome composition, particularly saprophytic microbes, with plant composition and diversity can result in higher decomposition rates (Mori et al., 2020; Veen et al., 2015; Hector et al., 2000) (Glassman et al., 2018; Podzikowski et al., 2024), potentially feeding back on productivity. Finally, diversity of mutualists – both rhizobia and AMF – have been shown to contribute to greater complementarity in plant productivity relationships via increasing plant access to essential nutrients (Magnoli and Bever, 2023). The differential sensitivities of these microbial groups to plant composition and diversity have major implications for the predominant direction of feedback on plant populations and communities, however this range of microbial groups have rarely been measured together.

To test the relative sensitivities of soil microbiome functional and taxonomic components to plant composition, plant biodiversity, precipitation and their interactions, we designed an experiment that independently manipulates plant species richness, plant family composition, and precipitation. While fungal pathogens, saprotrophs, AM fungi, bacteria, and oomycetes have all been shown to rapidly respond to gradients of plant community composition and diversity in the first growing season of this experiment (Burrill et al., 2023), it is unclear whether microbiome shifts in diversity and composition are expected to continue in the same direction over longer periods of time. In the current study, we re-sampled soils from this experiment to test whether the soil microbiome differentiated with plant composition and diversity in year three. In addition, here we test whether the microbiome responds to 50% or 150% of ambient rainfall treatments, as well as whether microbiome components respond to interactions between precipitation and plant composition and diversity.

## Methods

### Study system and experimental design

This experiment took place within the 250-ha Nelson Environmental Study Area (“NESA,” Kettle, 2016) at the University of Kansas Field Station, north of Lawrence, KS (39.052437, -95.191584). Historically, this land was inhabited by the Kansa and Osage peoples, who were forcibly displaced by European settlers. Many details of this piece of land history have been lost and/or destroyed. Our understanding is that the land that is now called NESA transitioned from native tallgrass prairie to cropland and pasture, ~100 years prior to acquisition by KU between 1970-1990. At the time of planting the experiment in 2018, the land was considered “post-agricultural,” dominated by established early cool season grasses (Kettle et al., 2000). Roughly 5 years prior to planting the experiment, the area where planting occurred had been abandoned following a rodent enclosure experiment, with the existing vegetation composed mostly of native warm season grasses. The mean annual cumulative precipitation at this site is 990 mm with about 70% of precipitation occurring during the growing season between April-September, and mean annual temperature 12.7 C (University of Kansas Field Station NEON).

The experiment was planted in 2018 and the soil microbiome was enriched by inoculation with soil from a nearby unplowed native prairie (Welda, KS) at the time of planting to reintroduce native microbes. A total of 240 plots were designed to include equal representation of 18 prairie plant species (6 from each plant family Asteraceae, Fabaceae, Poaceae). See **Supplementary Table S1** for more plant species names and life histories. The plant species richness treatment includes monoculture, 2, 3, and 5/6 species plots. In addition, these plots were either planted in phylogenetic under-dispersed (all within one plant family) or over-dispersed (plants from more than one family). The species combinations selected for each mixed plot were chosen at random without replacement, with the condition that each species was equally represented in every treatment combination (richness*phylogenetic dispersion*precipitation), yielding 36 monocultures, 36 two species plots, 24 three species plots and 24 six species plots within each of two precipitation treatments. This original design was then modified when one of the grass species failed to establish, yielding a final design of 38 monocultures, 36 two species plots, 20 three species plots, 8 five species pltos and 16 six species plots in each precipitation treatment. Finally, all plot combinations were replicated in paired shelters that received 50% or 150% ambient rainfall each growing season (**Supplementary Figure S1**). We prioritized a high level of replication and full factorial design, therefore the additional 50% resources to establish an ambient precipitation treatment was not feasible. We include each species in every treatment combination within each of two complete “blocks”, each of which are made up of three “subblocks” composed of paired rain exclusions that were randomly assigned to the two precipitation treatments. For more detailed information on the experimental design, refer to the supplemental document and **Supplementary Figure S1**.

In July 2020, 0.1 m x 0.1 m biomass strips were collected using electric shears from a standardized area in each plot. All species were keyed out, biomass dried, and weighed. Realized plant species proportions for all planted species were calculated for all plots by dividing the dried biomass of each planted species by the total plant dried biomass for each plot.

### Soil collection

In 2020, three soil cores taken to a depth of 20 cm were homogenized for each plot for analyses of the soil microbiome. Between plots, coring devices were rinsed and scrubbed of dirt in a water bucket, then sterilized in a bucket with 80% diluted ethanol. Samples were immediately placed in a large cooler with ice packs, then at the end of the day moved to a -20°C freezer (Delavaux et al., 2020). A subset of soil and roots (kept together, not separated) from each sample was weighed out to 0.25 g and placed into DNA extraction tubes. We extracted DNA from these samples using the Qiagen DNeasy PowerSoil kit. As we planned to compare 2020 samples to samples taken in 2018, our protocols mimicked those from our 2018 sampling, with two exceptions: in 2018, plots with identical species richness and composition treatments across sub-blocks were pooled and homogenized before DNA extraction, as the precipitation treatment was not fully operational until spring 2019 (Burrill et al., 2023), and roots and soil were sampled separately. For analyses that include both 2018 and 2020 data, we use soil DNA sequences from 2018 sampling.

### Microbial community library preparation

We sequenced amplicons targeting bacterial, fungal, oomycete, and AM fungal communities. For all communities, we used a two-step PCR process, with the first PCR reactions amplifying community-specific primers, and the second PCR binding unique barcode combinations using Nextera XT Index Kit v2 (Illumina, San Diego, CA, USA). Following each PCR step, sample products were checked on 1.5% (w/v) agarose gel to estimate the quality of PCR products and confirm the correct base pair length were amplified. Then, we performed a clean-up step for each PCR sample, using AMPure XP beads (Beckman Coulter, Brea, CA, USA). Prior to sending samples for sequencing, we measured PCR product concentration using an Invitrogen Qubit 3.0 Fluorometer (Thermo Fisher Scientific, Waltham, MA, USA). Adaptor ligation and sequencing was performed by an Illumina MiSeq v3 PE300 Next-Gen Sequencer at the Genome Sequencing Core (GSC), the University of Kansas.

For fungi, AM fungi, and bacteria, the first PCR used a mixture of 1 μl sample DNA, 10.5 μl ddH_2_O, 0.5 μl each of forward and reverse primer and 12.5 μl of Master Mix Phusion (Thermo Fisher Scientific, Waltham, MA, USA), for a total PCR volume of 25 μl. For these communities the second barcoding PCR used 5 μl cleaned up sample DNA from the first PCR, 10.5 μl ddH_2_O, 2.5 μl each of forward and reverse barcode primers, and 25 μl of Master Mix Phusion, for a total volume of 45 μl.

The primers used for fungi targeted the internal transcribed spacer (ITS) regions forward fITS7 (5’-GTGAGTCATCGAATCTTTG-3’) and reverse ITS4 (5’-TCCTCCGCTTATTGATATGC-3’) (Ihrmark et al., 2012). The first PCR cycle for fungi began at 94°C for 5 min, followed by 35x (94°C for 30 sec, 57°C for 30 sec, 72°C for 30 sec), 72°C for 7 min, ending on 4°C until retrieved from the thermocycler. The barcode PCR cycle began at 98°C for 30 sec, followed by 10x (98°C for 10 sec, 55°C for 30 sec, 72°C for 30 sec), 72°C for 5 min, ending on 4°C until retrieved from the thermocycler.

We used forward fLROR (5’-ACCCGCTGAACTTAAGC-3’) and reverse FLR2 (5’- TCGTTTAAAGCCATTACGTC-3’) primers to target the large subunit (LSU) region of AM fungi (House and Bever, 2018; Delavaux et al., 2022). The first PCR cycle for AM fungi began at 94°C for 5 min, followed by 35x (94°C for 30 sec, 48°C for 30 sec, 72°C for 30 sec), 72°C for 10 min, ending on 4°C until retrieved from the thermocycler. The barcode PCR cycle was the same as for fungi.

For bacteria, we used primers that targeted the V4 region of 16S small subunit (SSU) of ribosomal RNA, forward 515F (5’-GTGYCAGCMGCCGCGGTAA-3’) and reverse 806R (5’-GGACTACNVGGGTWTCTAAT-3’) (Parada et al., 2016). The first PCR cycle and the barcode PCR cycles were the same for bacteria as for fungi.

For oomycetes, we targeted ITS using forward ITS300 (5’-AGTATGYYTGTATCAGTGTC-3’) and reverse ITS4 (5’-TCCTCCGCTTATTGATATGC-3’). The first PCR used a mixture of 1 μl sample DNA, 17 μl ddH_2_O, 1 μl each of forward and reverse primer and 5 μl of HOT FIREPol (Solis Biodyne, Tartu, Estonia), for a total volume of 25 μl. We use HOT FIREPol, as it has been successful in amplifying oomycete DNA (Hunter et al., 2023), whereas we have had little success using Phusion to amplify oomycete DNA. The first PCR cycle for oomycetes began at 95°C for 15 min, followed by 35x (95°C for 30 sec, 55°C for 30 sec, 72°C for 1 min), 72°C for 10 min, ending on 4°C until retrieved from the thermocycler. The second barcoding PCR used 1 μl cleaned up sample DNA from the first PCR, 18 μl ddH_2_O, 0.5 μl each of forward and reverse barcode primers, and 5 μl of HOT FIREPol, for a total volume of 45 μl. The oomycete barcode PCR cycle began at 95°C for 15 min, followed by 35x (95°C for 30 sec, 55°C for 30 sec, 72°C for 1 min), 72°C for 10 min, ending on 4°C until retrieved from the thermocycler.

### Bioinformatics

We used the QIIME2 pipeline to process raw FASTQ data (Bolyen et al., 2019), including steps to demultiplex and remove primers, filter chimeras for quality control, de-noise and merge using *dada2* (Callahan et al., 2016). For quality control, we filtered out ASVs that only appeared 5 times or fewer across all samples. All communities were either open-reference clustered or blasted against taxonomic databases to identify ASVs. Taxonomy was assigned to all ribosomal sequence variants in QIIME2 using a feature classifier trained with the SILVA 99% database for bacteria (Quast et al., 2013) and the UNITE 99% database for fungi (Version 18.11.2018). For AM fungi LSU amplicons, we excluded non-AM fungi sequences by building a phylogenetic tree using the curated database of AM fungi (Krüger et al., 2012) using *Mortierella elongata* sequences as the outgroup in RAxML v8 (Stamatakis, 2014; House and Bever, 2018, Delavaux et al., 2021b, Delavaux et al., 2022). For oomycetes, we checked the identity of resulting ASVs against the NCBI oomycote ITS2 sequence database using the Basic Local Alignment Search Tool, BLAST v. 2.6.0 (Altschul et al., 1997), using default parameters. For the purposes of this study, we make the generalization that terrestrial oomycetes primarily act as parasites of vascular plants and analyses are interpreted as though oomycetes in our plots likely function as plant pathogens (Oliverio et al., 2020; Rojas et al., 2019).

To filter fungal ASVs into putative functional guilds, we used the FungalTraits database (Pölme et al., 2021). Fungal pathogens were filtered out if the ASVs “primary_lifestyle” was “plant_pathogen” or “unspecified_pathogen.” Fungal saprotrophs had “primary_lifestyle” of “litter_saprotroph,” “soil_saprotroph,” “wood_saprotroph,” and “unspecified_saprotroph.” Of 5346 identified fungal ASVs, 626 were putative pathogens and 1357 were putative saprotrophs. To filter rhizobial N-fixing bacteria, we subset out genera that typically act as N-fixers from the Silva taxonomy matches: *Bradyrhizobium, Ensifer, Mesorhizobium*, and those in the *Allorhizobium-Neorhizobium-Pararhizobium-Rhizobium* and *Burkholderi-Callabelleronia-Paraburkholderia* groups. From our original table with 3881 bacteria ASVs, 236 were subset out as rhizobia.

### Statistical analyses, 2020 sequencing data

All statistical analyses were done in R version 4.3.1. For both univariate and multivariate responses, we used two approaches to analysis: 1) using the full model enabled by our full factorial design and 2) using model selection approaches to identify the best model nested within that full factorial design. Generally these two approaches yielded similar interpretations, we note the few occasions where this was not true. We report the best model in the main text and the full model in the electronic appendix. For fungal pathogens and saprotrophs, we calculated relative abundance for each by the proportion of sequencing reads over total fungal sequencing reads. Relative abundance of rhizobium was similarly calculated as the proportion of rhizobium sequencing reads over total bacterial reads per sample. For each community, we calculated microbial diversity (H’) using the vegan package (Oksanen et al., 2019). We used the Shannon-wiener index, as it accounts for both richness and evenness, so that rare ASVs are more fully incorporated into the analyses. For both diversity and relative abundance response variables, we used *glmulti* to run Akaike Information Criterion (AIC) model comparisons (Calcagno, 2020) for generalized linear models with the following explanatory variables: block (see **Supplementary Figure S1**), the interaction of plant family composition, plant species richness, and precipitation treatments, and the proportion of each of the 17 plant species as covariates. We allowed the model comparisons to iterate 1000 times, at which point, a best model was selected with main effects. Finally, we ran linear mixed-effect models using *lme* (Bates et al., 2015) for each response variable using the selected models, with the interaction of subblock and precipitation as a random variable. To confirm that response to realized species proportions are not different from the response to the designed proportions, we also ran the full model for each microbial response using realized plant species proportions. We tested the correlation between residuals of both models for each microbial community (**Supplementary Table S2**).

To calculate microbial community composition, we first used the *transform* function with “robust center log-ratio” transformation for all ASV tables (Martino et al., 2019). Then, we used the *vegdist* function from the vegan package (Oksanen et al., 2019) to calculate the Aitchison distance between samples (Gloor et al., 2017; Martino et al., 2019). We did extensive literature review to select the most appropriate distance metric for these analyses. We found that microbial analyses are moving towards using robust center log ratio transformation in combination with Aitchison distances, rather than the previous methods of rarefying and Bray-Curtis distances, due to the inherent biases and statistical errors that arise with the large number of 0’s in ASV sequencing data (Martino et al., 2019). We first performed a redundancy analysis for each microbial community composition response to block, the interaction of plant family composition, plant species richness, and precipitation treatments, with the proportion of each of the 17 plant species as covariates, using a permutational multivariate analysis of variance (permanova) via the *rda* function in the vegan package (Oksanen et al., 2019). Similar to the linear responses, we then used the *ordistep* function (Oksanen et al., 2019) with 200 permutations to select the best fit model for each microbial group. We then report the main effects using *adonis2* (Oksanen et al., 2019). To measure the spread of each microbial community composition within samples, we ran the *betadisper* function (Oksanen et al., 2019) for precipitation and plant family treatments when there was a significant community response to those factors in the permanova. We then tested the differences in beta dispersion using an anova (**Supplementary Table S3**).

### Divergence from 2018 sampling

**Figure 1:**
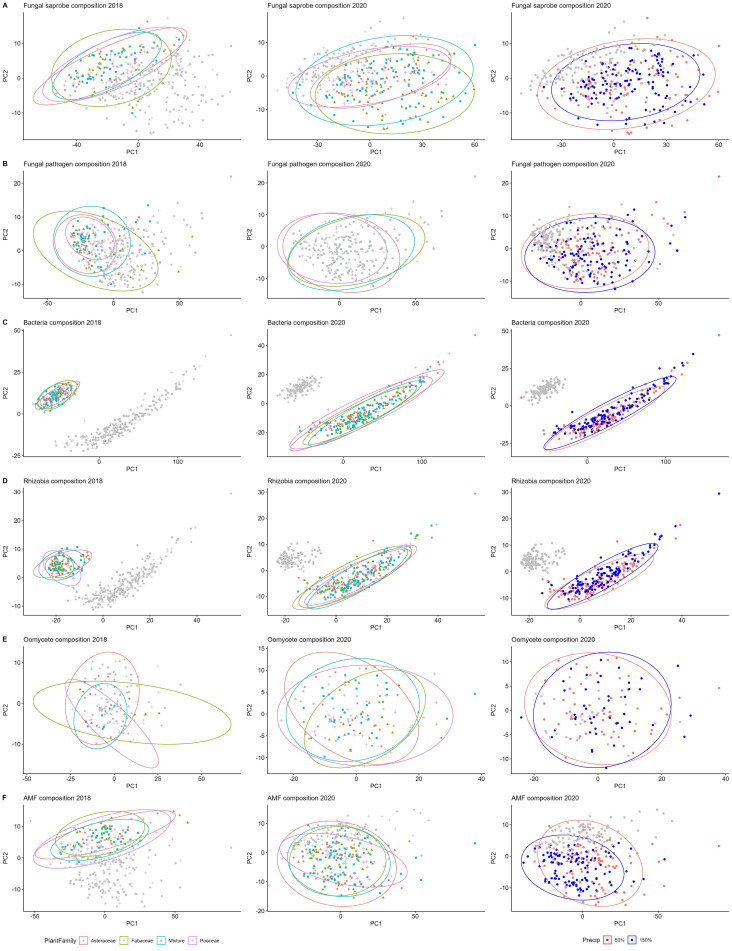
Microbial composition shifts from year 1 to year 3. Microbial composition among plant family treatments for year one (2018), with year three (2020) in grey (left); year three, with year one in grey (middle); composition between precipitation treatments in year three, with year one in grey (right). Right column shows 50% precipitation in red, 150% precipitation in blue, and 2018 data in grey for all. Fungal saprotrophs **(A)**, fungal pathogens **(B)**, bacteria **(C)**, rhizobia **(D)**, oomycetes **(E)**, and AM fungi **(F)**.

In order to visualize changes in microbial community composition between 2018 and 2020 soil communities, we re-ran bioinformatics pipelines for each community (fungi, oomycetes, all bacteria, rhizobia, and AM fungi) on the combined raw sequencing reads for both years. We then performed the same robust central log ratio transformation and calculated Aitchison distances for combined datasets by community, which were used to calculate principal component analyses. We emphasize here that we are not comparing these sampling years in statistical tests; we ran the bioinformatics and distance metrics together in pursuit of visualizing the differences on the principal component axes (**Figure 1**).

### Principal component analyses

**Figure 2:**
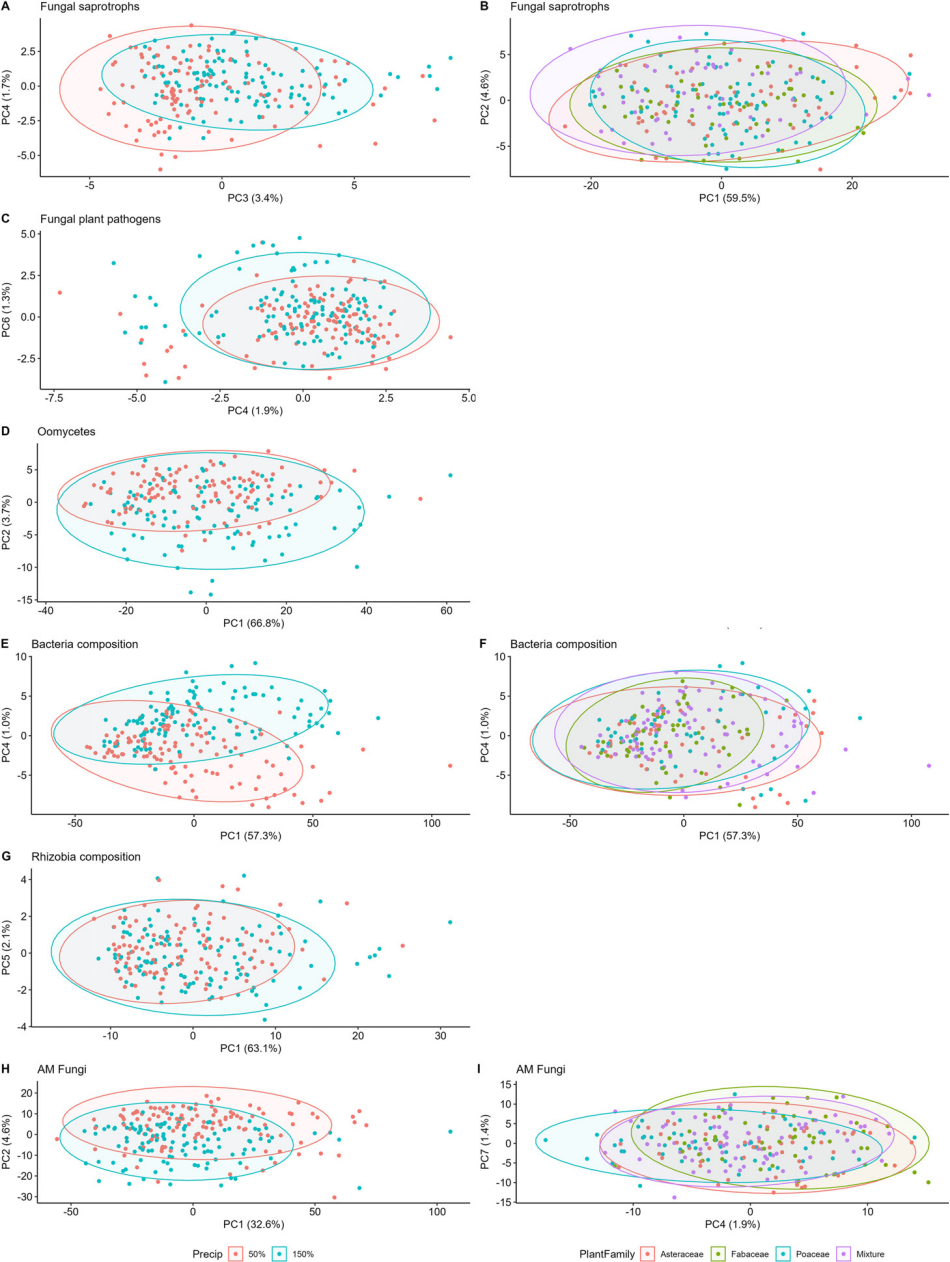
Microbial composition among plant family and precipitation treatments in year 3. Precipitation community differences in the left column, plant family differences on the right. Top row fungal saprotrophs **(A, B)**, fungal plant pathogens **(C)**, oomycetes **(D)**, bacteria **(E, F)**, rhizobial bacteria **(G)**, and AM fungi **(H, I)**.

For 2020 sampling data alone, we used the *prcomp* function to calculate principal component axis coordinates for each microbial community with center=T. We ran a generalized linear model on the first ten axes to identify axes that could illustrate significant differences in the permanova model output; predictor variables were the same in this glm as in permanova. The first two axes that differ significantly in the plant family composition and precipitation treatments were used to plot those communities (**Figure 2**). For 2018 and 2020 combined data, we used the same principal component calculations and plotted the variables using axes that had been significantly different for the 2020 data alone.

## Results

### Diversity

**Figure 3:**
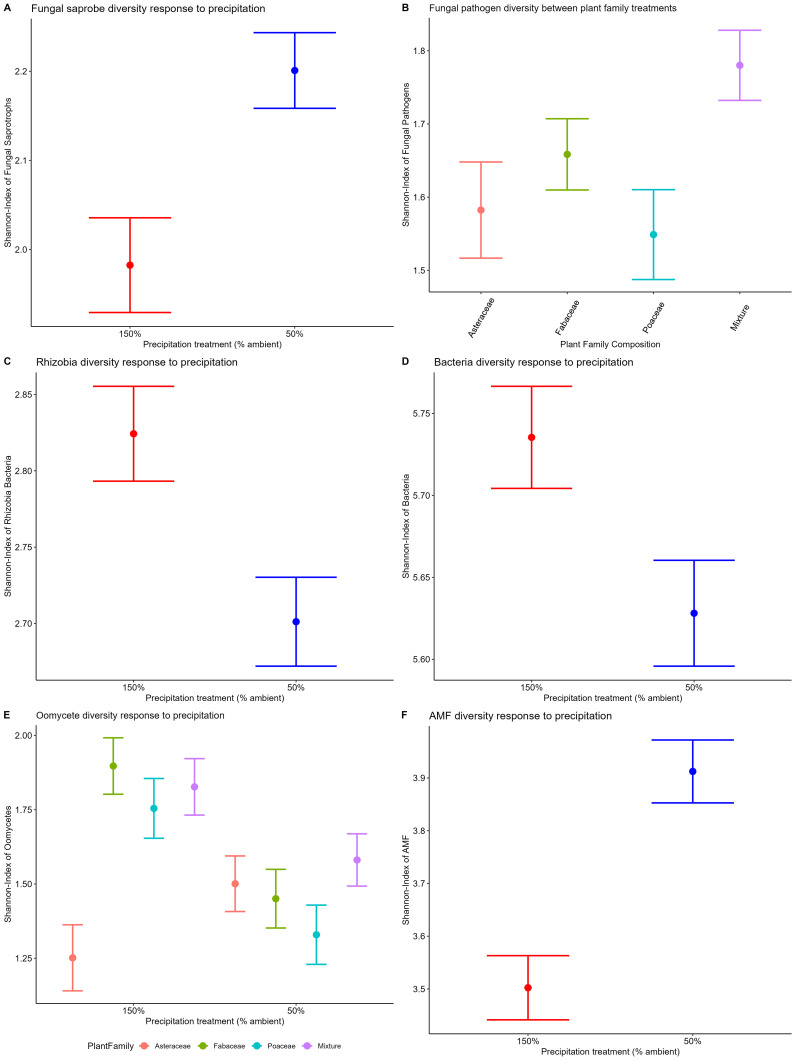
Shannon-Weiner diversity for each microbial community response to precipitation treatment and plant family composition. Fungal saprobe (**A**, p=0.01 precipitation), fungal pathogen (**B**, p=0.03 plant family), rhizobial bacteria (**C**, p<0.01 precipitation), non-rhizobial bacteria (**D**, p<0.01 precipitation), oomycetes (**E**, p=0.01 plant family), and AM fungi (**F**, p<0.01 precipitation). Oomycetes broken down by plant family composition treatment to visualize significant interaction between PlantFam*Precip in model output (**Table 1**).

We found the Shannon-Weiner diversity of fungal saprotrophs (p=0.016) and AM fungi (p<0.001) to respond significantly to precipitation manipulation (**Table 1**). Fungal saprotroph and AM fungal diversities were higher in the 50% ambient precipitation treatment (**Figures 3A**, **F**, respectively), while bacteria and oomycete diversities were higher in the 150% ambient precipitation treatment (**Figures 3C, D**, p>0.05, **Figure 3E**, p>0.05, respectively). Fungal pathogen diversity did not differ between precipitation treatments, but did between plant family composition (p<0.05 =, **Table 1**; **Figure 3B**). Oomycete diversity was higher in Fabaceae single-family and mixture plots, and lowest in Asteraceae single-family plots, with Poaceae single-family plots intermediate (p<0.05, **Table 1**, **Supplementary Figure 1C**). Plant species richness did not have a direct effect on diversity of any individual microbial group (**Table 1**). In the full model (**Supplementary Table S2**), there was a significant difference of fungal pathogen diversity to plant family (p=0.03, **Figure 3B**). Additionally, in the full model oomycete diversity differed between the interaction of plant family and precipitation (p=0.01, **Figure 3E**).

**Table 1:** Mixed effects model outputs for each microbial Shannon-Weiner diversity response to the planting design.

Diversity		
Saprotrophs	Std. Error	p-value
intercept	0.069	0.000E+00
Precip	0.091	**0.016**
ELYCAN	0.094	**0.040**
BOUGRA	0.092	0.106
DALPUR	0.092	**0.053**
Pathogens	Std. Error	p-value
intercept	0.163	0.000E+00
Block	0.098	0.177
PlntFamFAB	0.078	0.174
PlntFamMIX	0.075	**0.0003**
PlntFamPOA	0.090	0.397
BOUGRA	0.078	0.125
PANVIR	0.083	0.227
Oomycetes	Std. Error	p-value
intercept	0.116	0.000E+00
PlntFamFAB	0.089	**0.003**
PlntFamMIX	0.085	**0.0004**
PlntFamPOA	0.095	**0.019**
Precip	0.143	0.104
ANDGER	0.088	0.148
Bacteria	Std. Error	p-value
intercept	0.149	0.000E+00
Block	0.089	**5.828E-06**
Precip	0.089	0.233
AMOCAN	0.046	**0.060**
DALCAN	0.046	**0.051**
Rhizobia	Std. Error	p-value
intercept	0.004	0
Block	0.003	**6.05E-09**
Precip	0.003	0.608
BOUGRA	0.002	0.692
DALPUR	0.002	**0.008**
AMF	Std. Error	p-value
intercept	0.275	0.000E+00
Block	0.165	0.347
Precip	0.165	**0.014**
AMOCAN	0.118	0.867
DALCAN	0.117	0.234

Predictors are from best fit model for each group. “PlntFamFAB” is plant family composition with Fabaceae only, “PlntFamMIX” are mixtures, and “PlntFamPOA” are Poaceae only. “Precip” is precipitation treatment 50% ambient rainfall. Full model outputs in **Supplementary Table S2**.

P values <0.1 are in bold to emphasize significant and marginally significant responses.

### Relative abundance

**Figure 4:**
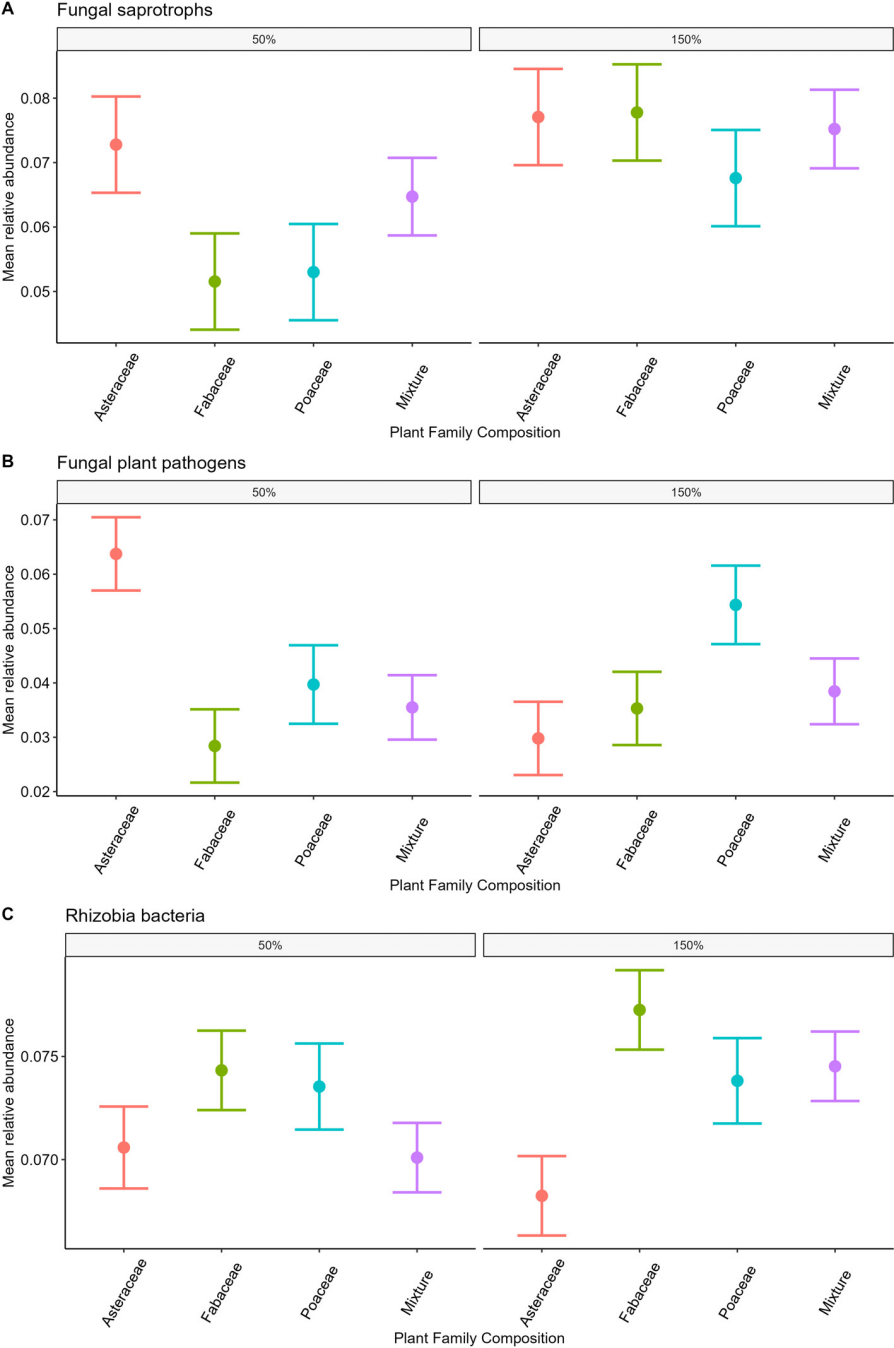
Relative abundance of fungal pathogens, saprotrophs, and rhizobial bacteria among plant family and precipitation treatments. Mean relative abundance of fungal saprotrophs (**A**, p>0.05), pathogens (**B**, p<0.05) and rhizobial bacteria (**C**, p<0.05) between plant family treatments. Differences between precipitation treatments also shown, fungal saprotroph relative abundance higher in 150% precipitation (**A**, p<0.05 =) and fungal plant pathogen relative abundance had an interaction between plant family and precipitation in the full model (**B**, **Supplementary Table S3** p<0.01).

The relative abundance of fungal saprotrophs was highest in the 150% ambient precipitation plots (p<0.05 =, **Table 2**, **Figure 4A**). Fungal pathogen relative abundance did not differ between precipitation treatments alone, however there was a significant differences in pathogen relative abundance between plant family treatments, highest in Asteraceae only plots in 50% precipitation and Poaceae only plots in 150% (p<0.01, **Table 2**, **Figure 4B**), as well as an interaction between plant family and precipitation in the full model (p<0.01, **Supplementary Table S3**, **Figure 4B**). Rhizobial bacteria relative abundance was highest in plots with only Fabaceae and lowest in plots with only Asteraceae (p<0.05, **Table 2**, **Figure 4C**), though there was no significant difference between precipitation treatments.

**Table 2:** *Relative abundance of functional guilds.* Generalized linear model response of relative abundance for fungal saprotrophs, pathogens, and rhizobial bacteria to the full planting model.

Relative Abundance		
Saprotrophs	Std. Error	p-value
intercept	0.010	4.665E-11
Block	0.006	0.096
PlntSpRich	0.002	**0.026**
Precip	0.006	**0.017**
ANDGER	0.007	0.087
ELYCAN	0.007	**0.0002**
DALPUR	0.007	**0.021**
DESILL	0.007	**0.001**
CORTIN	0.008	**0.001**
Pathogens	Std. Error	p-value
intercept	0.005	0.000E+00
PlntFamFAB	0.007	**0.023**
PlntFamMIX	0.006	**0.049**
PlntFamPOA	0.007	0.314
BOUGRA	0.007	0.105
Rhizobia	Std. Error	p-value
intercept	0.004	0.000E+00
Block	0.002	2.757E-09
PlntFamFAB	0.002	**0.008**
PlntFamMIX	0.002	**0.032**
PlntFamPOA	0.002	**0.0004**
ANDGER	0.002	**0.007**
DALPUR	0.002	**0.048**

Predictors are from best fit model for each group. “PlntFamFAB” is plant family composition with Fabaceae only, “PlntFamMIX” are mixtures, and “PlntFamPOA” are Poaceae only. “Precip” is precipitation treatment 50% ambient rainfall. “PlntSpRich” is realized plant species richness. Full model outputs in **Supplementary Table S3**.

P values <0.1 are in bold to emphasize significant and marginally significant responses.

### Composition

All components of the soil microbial community, including fungal saprotrophs, fungal pathogens, oomycetes, bacteria, and AM fungi, differentiated with precipitation (p<<0.05, **Table 3**). In addition, bacteria and AM fungal composition differentiated between plant family composition treatments (p <0.05). Fungal pathogen composition differed between plant family treatments in the full model (p<0.01, **Supplementary Table S4**, **Figure 2B**), but not oomycetes. Fungal saprotrophs were the only group to differentiate between plant species richness treatments (p=0.02 in full model **Supplementary Table S4**, but p>0.05 in reduced model, **Table 3**). Fungal pathogen, saprotroph, and oomycete compositions did not differ between planted species. Bacterial composition differed with two grass species and an aster, and surprisingly rhizobial bacteria composition had no significant response to any individual legume species. AM fungal composition differed significantly with one legume species. There were no significant differences in microbiome composition for any community in response to interactions of precipitation, plant species richness or family composition; though oomycetes had a marginal difference between interaction of plant family and precipitation treatments (p=0.06, **Table 3**).

**Table 3:** Permanova table, community response.

Saprotrophs	Df	R2	Pr(>F)
PlntSpRich	1	0.004	0.651
Precip	1	0.014	**0.001**
DALCAN	1	0.005	0.282
EUPALT	1	0.004	0.397
Residual	230	0.973	
Total	234	1	
Pathogens	Df	R2	Pr(>F)
Block	1	0.023	**0.001**
PlantFam	3	0.012	0.494
Precip	1	0.007	**0.003**
PlantFam* Precip	3	0.014	0.208
Residual	226	0.943	
Total	234	1	
Oomycetes	**Df**	**R2**	**Pr(>F)**
Block	1	0.012	**0.001**
PlantFam	3	0.013	0.702
Precip	1	0.013	**0.001**
PlantFam* Precip	3	0.015	**0.064**
Residual	209	0.947	
Total	217	1	
Bacteria	Df	R2	Pr(>F)
Block	1	0.034	**0.001**
PlantFam	3	0.014	**0.058**
Precip	1	0.012	**0.001**
BOUGRA	1	0.005	**0.023**
PANVIR	1	0.005	0.157
LIAPYC	1	0.005	**0.056**
Residual	224	0.926	
Total	232	1	
Rhizobia	Df	R2	Pr(>F)
Block	1	0.022	**0.001**
Precip	1	0.008	**0.004**
AMOCAN	1	0.004	0.506
Residual	228	0.966	
Total	231	1	
AMF	**Df**	**R2**	**Pr(>F)**
Block	1	0.018	**0.001**
PlantFam	3	0.017	**0.001**
PlntSpRich	1	0.004	0.615
Precip	1	0.018	**0.001**
DALCAN	1	0.004	0.173
DESILL	1	0.006	**0.001**
LIAPYC	1	0.004	0.152
CORTIN	1	0.005	0.119
Residual	224	0.924	
Total	234	1	

Aitchison distance matrices for each community ran in a permanova with the best fit models from ordistep model selection. Full model outputs in **Supplementary Table S4**.

P values <0.1 are in bold to emphasize significant and marginally significant responses.

Overall, the greatest differences in beta dispersion were between plant family treatments for fungal saprotrophs, where Asteraceae-only plots had the most similar composition (p=0.024, **Supplementary Table S3**, **Supplementary Figure S2**). Differences in beta dispersion of fungal pathogens and saprotrophs was not detected in response to precipitation, however, oomycete beta dispersion was marginally different between precipitation treatments (p=0.06, **Supplementary Table S2**, **Supplementary Figure S2**). Beta dispersion in composition of AM fungi (p=0.04) and rhizobial bacteria (p=0.037), but not all bacteria, were also significantly different between precipitation treatments.

## Discussion

Across all functionally distinct groups of soil bacteria, fungi, and oomycetes, we found highly significant responses of diversity and/or composition to precipitation treatments, with precipitation explaining more variation than plant composition for both bacteria and oomycetes (see R² in permanova **Table 2**). We saw shifts in microbial diversity, as AM fungal and fungal saprotroph diversity decreased with precipitation, while diversity of oomycetes and bacteria generally increased. Fungal saprotroph and AM fungal composition differentiated significantly between plant family composition treatments, with marginal differences in bacterial composition (**Table 2**). In general, both plant family composition and species richness in combination with precipitation treatments had independent impacts on microbiome composition. The one exception is oomycetes, which have moisture dependent life history stages and, interestingly, showed stronger differentiation between families in high precipitation. These plant composition effects on microbial composition likely feed back on plant community structure and function, as was observed in year one (Wang et al., 2023). The contrasting sensitivities of microbial groups to variation in precipitation suggest that there could be critical shifts in microbial functions with climate change.

### Guild responses to plant community composition

#### Mutualists

Of the two mutualist microbial groups we measured, both AM fungi and rhizobia were responsive to the planting design. First, AM fungal communities differentiated with plant family composition in the third year sampling, despite finding no difference in year one (Burrill et al., 2023). This suggests that AMF response to host plant species takes more time than other microbiome components. Our findings support that host specificity of AM fungal growth rates found in greenhouse studies (Bever et al., 1997, Mangan et al., 2010a) also apply to the field, but that it takes greater than 4 months, and up to three years for such divergence to be detected. AM fungal community compositional differences between plant family treatments also suggest a substantial phylogenetic component of AM fungal response to host species. Of all microbial components, AM fungal composition was most sensitive to the planted proportions of individual species (**Table 3**), indicating host specific differences in AM fungal growth rates. Accumulating research on AM fungi shows that, while they often have low specificity in association with plant hosts, they can have high specificity in their impact on and response to individual plant species (Bever et al., 1997, Bever, 2002; Mangan et al., 2010a; Martínez-García and Pugnaire, 2011, Koziol and Bever 2016). Additionally, four of the six plant species exerting detectable impacts on AM fungal composition are late successional plants, which have been found to be more sensitive to AM fungal identity than early successional species (Cheeke et al., 2019, Koziol and Bever 2016). This could feedback on host fitness, potentially influencing plant species coexistence or succession (Bever, 1999, 2002; Koziol and Bever, 2019), though further work is required to test these possibilities.

Rhizobia bacteria were also responsive to the planting design, particularly in relative abundance, which was highest in Fabaceae only plots, as expected due to the legume-rhizobia symbiosis. Prior work found significant positive feedback among the legume species used in this experiment, likely driven by rhizobial mutualists (Wang et al., 2023). However, despite known specificity in legume-rhizobia associations (Andrews and Andrews, 2017), we did not detect composition or diversity responses to legume species or legume-only plots (**Tables 1**, **3**). There is surprisingly little work on the relationship between legume and rhizobial diversity, with a general lack of knowledge of rhizobia communities outside of nodules (Miranda-Sánchez et al., 2016). Further work on drivers of rhizobial diversity is needed to form better predictions about how these microbial communities may respond to changes in plant diversity and composition.

#### Pathogens

While both fungal pathogen and oomycete diversity responded to plant species richness in year one (Burrill et al., 2023), by the third year both pathogenic groups’ diversity had greater responses to the plant family composition treatment. Fungal pathogen diversity was highest in plant family mixtures (**Figure 3B**), suggesting the presence of multiple family-specific pathogens. These results are consistent with phylogenetic structure of plant pathogen specialization (Gilbert and Webb, 2007). Moreover, this host-specialization of pathogens can drive negative plant-soil feedbacks (Crawford et al., 2019) and plant species coexistence (Bever et al., 2015). Pathogen specialization can also generate pathogen dilution in mixture, as was found for fungal pathogens year one, with direct consequences for productivity gains with plant diversity (Burrill et al., 2023; Wang et al., 2023). Further support for dilution of fungal pathogens is observed, with their relative abundance being highest in single-family Asteraceae and Poaceae plots, compared to Fabaceae or family-mixtures (**Table 2**, **Figure 4B**).

Despite their pathogenic functionality in terrestrial systems, these data show a more complicated response of oomycetes than general host specificity. Oomycete diversity was relatively lower in plots with only Asteraceae (**Table 1**, **Supplementary Figure S1C**), which resonates with the first year pattern, in which oomycete diversity did not differ between Asteraceae-only plots and mixtures (Burrill et al., 2023). In the first year, oomycete composition had the greatest divergence in Fabaceae single-family plots, but two years later this pattern was muted. Here we find oomycete composition did not significantly differentiate between plant family composition treatments, despite differences with planted proportion of two aster and one grass species (**Table 3**). However, there was a noteworthy response to the interaction of precipitation and plant family composition treatments, with single-family divergence appearing to be stronger in the 150% precipitation treatment (**Supplementary Figure S3B**). The dependence of oomycete composition and diversity on the plant composition-precipitation interaction is consistent with expectations that oomycete pathogen specialization on their hosts would be enhanced by high precipitation treatments due to their life history. Differences in oomycete composition between monoculture plots have been shown to predict negative pairwise feedback and overyielding in mixture (Wang et al., 2023), which complements evidence of oomycete pathogens driving negative feedbacks that mediate plant species coexistence (Mills and Bever, 1998; Mangan et al., 2010b; Bever et al., 2015).

#### Saprotrophs

Fungal saprotroph and bacteria – major drivers of plant litter decomposition – both differed in composition between plant family composition treatments. Though we lack information to assign saprotrophic functionality to bacteria, many soil bacteria contribute to decomposition, so we consider their response here to potentially follow similar expectations as for fungal saprotrophs. Fungal saprotroph composition was particularly different in Poaceae and Asteraceae only plots (**Figure 4A**), but significant differences were also detected with three Fabaceae species (**Table 3**). Such differentiation of saprotrophs with plant family and species composition is consistent with these groups contributing to phylogenetic structure of home field advantage in decomposition, as observed by Podzikowski et al. (2024). We see stronger plant compositional impacts on soil saprotroph composition over time, suggesting the additional development of stronger home field advantage. Differences between bacterial community composition amongst plant family treatments were detected in the roots in year one (Burrill et al., 2023), and we continued to see this in year three. However, the significance of plant family was dampened in the third year, perhaps due to higher variation explained by precipitation treatment effects (**Table 3**). In the 50% precipitation treatment, bacterial diversity had a positive response to plant species richness in Asteraceae single-family plots, with a similar trend in family mixtures, but the opposite effect in Fabaceae and Poaceae single-family plots (**Supplementary Figure 1B**). This may further contribute to observed increases in decomposition rates in the 150% treatment (Podzikowski et al., 2024) and to decomposer bacteria home-field advantage (Austin et al., 2014; Veen et al., 2015).

### Guild responses to precipitation treatment

There were significant differences in diversity of most microbial components to precipitation treatments, 50% and 150% of ambient rainfall. AM fungal diversity increased in 50% ambient precipitation, which may reflect greater reliance of prairie plants on AM fungi for nutrient acquisition under dry conditions (Schultz et al., 2001, Auge, 2001). In addition, AM fungal community composition was significantly different between precipitation treatments. This shift in composition could benefit plant growth in dry environments, as drought adapted AM fungi have been shown to improve drought tolerance of their hosts in western US grasslands (Stahl and Smith, 1984). We also see that the variation in AM fungal composition between plots in the low precipitation treatment is greater than that between plots in the high precipitation treatment (**Supplementary Table S2**). While it is possible that this beta dispersion could result from greater sensitivity or specialization of the AM fungi and plant species combinations in dry conditions, this possibility is not supported by evidence of an interactive influence of plant composition and precipitation on AM fungal composition. Further exploration necessitates specific greenhouse tests of the ability of AM fungi from dry treatments to confer drought tolerance.

In contrast, oomycetes were found to have the opposite response to precipitation treatments. Specifically, oomycete diversity increased with high precipitation, consistent with observed oomycete diversity patterns in remnant prairies across the natural rainfall gradient from central Kansas to Illinois (Delavaux et al., 2021a). However, in contrast to field patterns observed in this same gradient, we did not observe changes in fungal pathogen diversity in response to the precipitation treatment. Nonetheless, oomycete responses are consistent with the high precipitation treatment facilitating more pathogenic microbes, likely since they use flagella as their mode of movement, and require water for sexual reproduction and transmission. We also observed greater variation in oomycete composition between plots within the high precipitation treatments (**Supplementary Table S6**). Further, with increased diversity of oomycetes in the high precipitation treatment, there may also be an increased occurrence of host specialization, which is further supported by the interaction of family composition and precipitation on oomycete composition. Together these results suggest that oomycete pathogens will be more important to plant species coexistence and overyielding as precipitation increases.

Bacteria and fungal saprotrophs were found to have opposite responses to precipitation manipulations. Soil bacterial diversity increased with high precipitation (**Figure 3D**). This is consistent with previous work showing soil bacteria are more sensitive to drought than fungi (de Vries et al., 2018). Given that plant decomposition rates have been observed to increase in the 150% precipitation treatment (Podzikowski et al., 2024), it is possible that increased bacterial diversity contributes to the observed increase in decomposition rates. Contrary to the bacterial response, we detected higher fungal saprotroph diversity in the 50% ambient precipitation (**Figure 3A**). This result is consistent with prior research showing increased fungal diversity under low rainfall (Hawkes et al., 2011). In addition, while fungal life history traits may be more conducive to wetter environments, fungi are also more capable of surviving during periods of extreme drought via dormant spore structures (Furze et al., 2017; Barnard et al., 2013). Together, these results may indicate relative shifts in contribution of bacterial and fungal decomposers, depending on precipitation conditions.

## Conclusions

We observed significant responses in functionally distinct groups of soil bacteria, fungi and oomycetes to precipitation and family composition treatments three years after planting, indicating that both plant community composition and precipitation matter, with relatively little interaction. Contrasting responses of mutualist AM fungi and pathogenic oomycetes to precipitation potentially indicate both AM fungal-mediation of plant drought tolerance and increased oomycete pathogen effects in high rainfall. Together these results suggest a negative directional shift in the soil microbiomes impact on plant fitness with increasing precipitation. Additional contrasts between fungal saprotrophs and bacteria suggest differential shifts in decomposer taxa, depending on climate conditions. Further, bacterial and fungal saprotroph differentiation with plant composition support prior research indicating that decomposers may have plant species litter preferences, thus driving home-field advantage in decomposition rates. Rapid responses of pathogens in year one (Burrill et al., 2023) were sustained in year three. Moreover, we find that oomycete specialization may be enhanced with precipitation. While we have direct evidence of fungal pathogen and oomycete specialization generating negative feedback on plant fitness and overyielding in mixtures (Wang et al., 2023), further investigations may elucidate whether other observed shifts in microbial communities influence terrestrial ecosystem functions. Interestingly, we did not find an interactive effect of precipitation treatments with plant species richness, instead demonstrating that microbial communities were independently affected by each of these factors. Together, our findings demonstrate the relative shifts of differential microbial functional groups in response to both plant community structure and climate, which likely have downstream impacts on terrestrial ecosystems under biodiversity loss and climate change.

## Data Availability

The datasets presented in this study can be found in online repositories. The names of the repository/repositories and accession number(s) can be found below: https://www.ncbi.nlm.nih.gov/, PRJNA1006419. Data analysis, statistics, and figures code https://github.com/Hburrill/FungPath-PrairiePlants/blob/63fa8b1d6dc0516a55eba4efc6d978251e23f8af/4A_2020_FrontiersFinal_Jan2025.R.
