# Supplementary material for: Soil microbiome composition is highly responsive to precipitation and plant composition manipulations in a field biodiversity experiment
Source: Front Microbiomes. 2025 Jan 31;4:1460319. doi: 10.3389/frmbi.2025.1460319 (PMC12993530; doi:10.3389/frmbi.2025.1460319)
Supplement: Supplementary file 1 [file DataSheet1.docx]

*Experimental design*

This experimental design focuses on three main treatments – plant species richness (monoculture, 2, 3, 5, or 6 species), plant family composition (Asteraceae only, Fabaceae only, Poaceae only, or a mixture of families), and precipitation (50% or 150% ambient cumulative rainfall during growing seasons). At the establishment of this experiment, 18 native prairie plant species were planted in a full-factorial design, with over-representation of monocultures, for a total of 240 plots. These plots were divided into 6 sub-blocks, within which are 2 rain shelter houses, each containing 20 plots (1.5 x 1.5 m, separated by 0.5 m margins in all directions). Within each shelter are 6 plots each of monoculture and 2-species combinations, 4 plots each with 3- and 6-species combinations. The 2 shelters within sub-block have plots with the same diversity and plant composition treatments and arrangement, with one replicate receiving 50% ambient rainfall and the other receiving 150% ambient rainfall within the growing season (Figure S1).


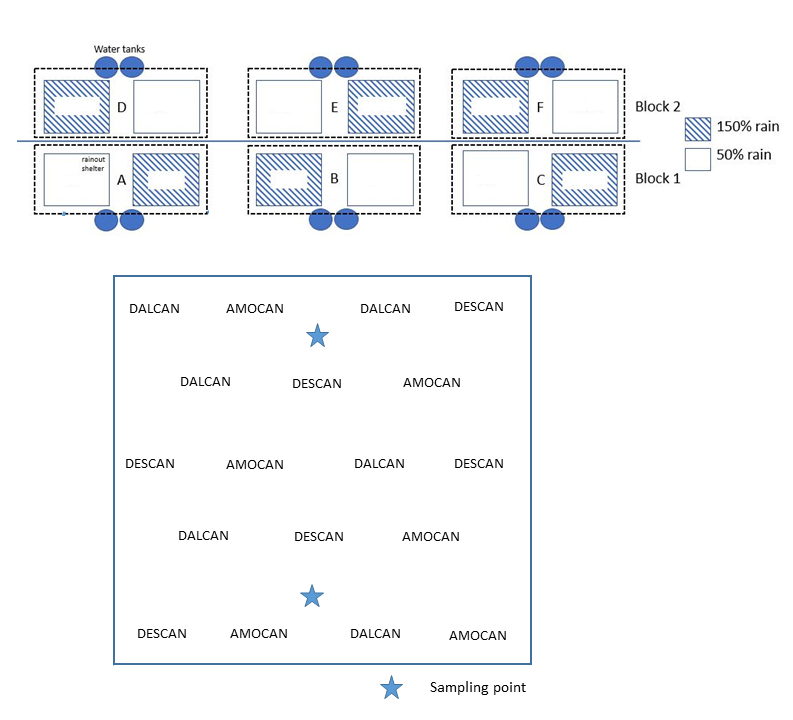


Figure S1 – Experimental plot layout, with Blocks 1 & 2 separated by a dirt road large enough for a small tractor. Subblocks (A-F) have replicate planting design, with 20 1mx1m plots per rainout shelter. Each paired subblock design received rainfall manipulations (50% or 150% ambient rainfall) each growing season, beginning 2019. Starred sampling points represent the unbiased soil core collection locations for each plot. The soil cores were pooled and homogenized for each plot, prior to weighing out the 0.25 g needed for DNA extraction.

To ensure precipitation treatments accurately represent rainfall treatments, rain-shelters were built with troughs to direct runoff into 3,000-gallon tanks at the edge of each house. Precipitation treatments were administered when wind was low, incrementally as ambient precipitation occurred through the growing season, via irrigation sprinklers. Sprinklers were calibrated for each shelter by collecting irrigation in 12 jars distributed in aisles throughout the shelter for 10 minutes, summing the total volume collected, and taking the average irrigation rate per house. This was then applied to each house in calculating amount of water to apply, depending on the treatment (50% or 150% ambient x calibration rate). Precipitation treatments were initiated at the end of the 2018 growing season, and not fully implemented until the 2019 growing season and continued each year. Rainfall exclusion tarps were removed after biomass was removed at the end of each growing season, thus precipitation treatments were only administered during April-October, and in the remaining months, all plots naturally received equal amounts of ambient rainfall. In 2018, all plots received ambient precipitation – 415.9 cumulative ambient precipitation April-October, as shelter exclusions had not been applied yet. In the growing season of 2019, ambient cumulative precipitation was 963.7 mm, 595.9 mm in 2020, 923.0 mm in 2021, and 691.3 mm in 2022.

Seeds were purchased from Hamilton Native Outpost, Stock Seed, Missouri Wildflowers, and Prairie Moon, which include cultivars native to the eastern Kansas/ western Missouri region. Plug plants were grown via sowing seeds into flats with autoclaved sterile potting soil and placing in cold-moist stratification for 4 weeks prior to germination. Once seedlings were mature enough to transplant, we planted them into Stewe and Sons groove tubes (GT51D) with 98 mL of whole-soil inocula from an unplowed native prairie near Welda, KS, which was acquired due to a highway widening development (38.182812, -95.261736). Plugs continued to grow in the greenhouse for 5 weeks prior to planting. During this time, the soil at the field site was tilled to a depth of 15.24 cm and an average 3.81 cm of soil from the native prairie was added to the surface of each plot.

At experiment establishment, a total of 18 plugs were planted in each plot, spaced evenly and mixing the plot’s assigned plant species randomly in a hexagonal array. In addition to planting plugs, plots were seeded at the time of planting in May 2018, and then each subsequent year in either January or February. For each plot species treatment, seed for each species was equally represented by pre-weighing 100 seeds for each species and evenly dividing the final mix of species by weight, resulting in 1800 seeds per blend. In addition to seeding each year, plot experimental design was also supported by annual weeding of non-planted species. In 2018 one round of weeding was accomplished, removing all forbs but leaving any grasses below 3 inches in height. In May, and again in June, for 2019-2022, two rounds of extensive weeding were accomplished, removing all non-planted species detected.

The species used in this experiment include 6 Asteraceae, 6 Fabaceae, and 6 (later, 5*) Poaceae. Species in Asteraceae were *Liatris pycnostachya, Coreopsis tinctoria, Echinacea pallida, Eupatorium altissimum, Silphium integrifolium,* and *Helianthus mollis.* Fabaceae species were *Amorpha canescens, Dalea candida, Dalea purpea, Desmanthus illinoensis, Desmodium canadense,* and *Chamaecrista fasticulata.* Poaceae species were *Schizachrium scoparium Andropogon gerardii, Elymus canadense, Bouteloua gracilis, Panicum virgatum,* and **Koeleria macrantha.* (*Note: One grass species, *K. macrantha* failed to establish and, in 2020, it was removed from the experiment via exclusion from re-seeding efforts and statistical analyses.) The selected plant species include a mix of early and late successional plants, varying in flowering time and height, and all but two species are perennial (S Table 1). Selected grass species also include both C3 and C4 photosynthesizers, which have been found to have higher water use efficiency (WUE). In addition, plants within Fabaceae form symbioses with *Rhizobia* bacteria in their root nodules. These microorganisms “fix” atmospheric nitrogen (N2) into an accessible form for plants, ammonia. For this reason, the presence of legumes have been identified as beneficial to other plant functional groups as well, since nitrogen is commonly a (co-) limiting nutrient for plants. Finally, amongst the asters in the experiment are *E. pallida,* which produces chemicals that are of medicinal use for humans (Percival 2000), as well as *S. integrifolium,* which is being studied for its potential as a more sustainable perennial crop alternative to sunflower oil (Peni et al. 2020, Van Tassel et al. 2017).

Table S1 – List of experiment species, their family, flowering time, height, life history strategy, photosynthesis method, and whether they have symbioses that help fix Nitrogen. Details found on Missouri Botanical Garden Plant Finder.


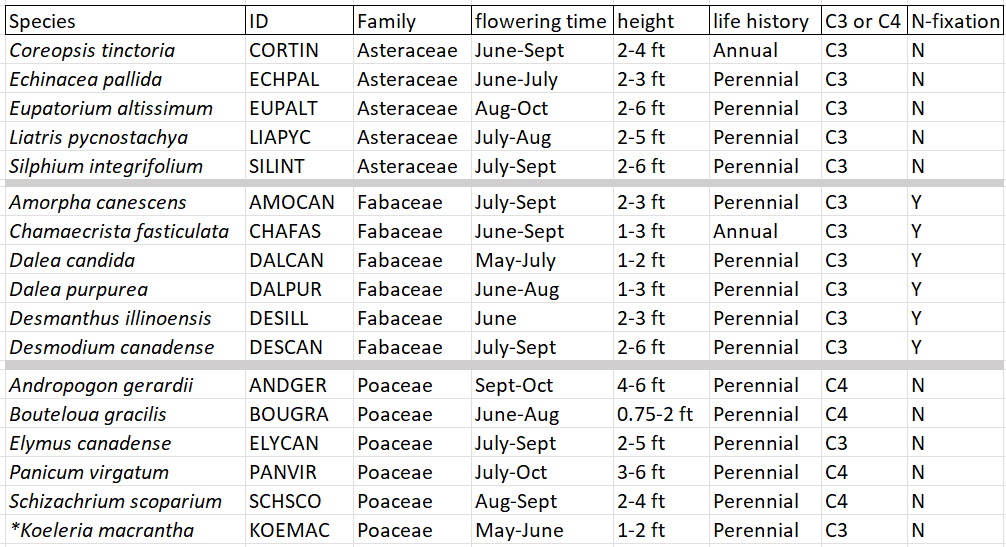


References

Peni D, Stolarski MJ, Bordiean A, Krzyżaniak M, Dębowski M. Silphium perfoliatum—A Herbaceous Crop with Increased Interest in Recent Years for Multi-Purpose Use. Agriculture. 2020 Dec;10(12):640.

Percival SS. Use of echinacea in medicine. Biochemical Pharmacology. 2000 Jul 15;60(2):155–8.

Van Tassel DL, Albrecht KA, Bever JD, Boe AA, Brandvain Y, Crews TE, et al. Accelerating Silphium Domestication: An Opportunity to Develop New Crop Ideotypes and Breeding Strategies Informed by Multiple Disciplines. Crop Science. 2017;57(3):1274–84.

*Supplemental tables and figures*

Table S2- **Microbial community Shannon-Weiner diversity response to planting design.** Generalized linear model output for each community diversity response to the planting design. “PlantFam” is plant family composition – single family (Asteraceae, Fabaceae, Poaceae), or Multi-family. “SpRich” is plant species richness, “Precip” is precipitation treatment 50% or 150% ambient rainfall. Plant species codes represent the planted proportion of each species.

| Diversity (H') | | | Fungal saprobes | | | Fungal pathogens | | Oomycetes | | Rhizobia | | Bacteria | | AMF | |
| --- | --- | --- | --- | --- | --- | --- | --- | --- | --- | --- | --- | --- | --- | --- | --- |
|  | **Df** | | | **F val** | **Pr>F** | **F val** | **Pr>F** | **F val** | **Pr>F** | **F val** | **Pr>F** | **F val** | **Pr>F** | **F val** | **Pr>F** |
| Block | | 1 | 2.80 | | 0.10 | 6.55 | 0.01 | 0.12 | 0.73 | 20.4 | <0.01 | 133.9 | <0.01 | 3.30 | 0.07 |
| **PlantFam** | | 3 | 0.77 | | 0.51 | 3.09 | **0.03** | 4.24 | **0.01** | 0.57 | 0.63 | 0.49 | 0.69 | 0.05 | 0.99 |
| **SpRich** | | 1 | 0.20 | | 0.66 | 0.11 | 0.74 | 0.28 | 0.60 | 0.49 | 0.48 | 0.48 | 0.49 | 0.02 | 0.90 |
| **Precip** | | 1 | 7.64 | | **0.01** | 0.56 | 0.45 | 12.8 | **<0.01** | 9.16 | **<0.01** | 8.88 | **<0.01** | 22.92 | **<0.01** |
| ANDGER | | 1 | 2.24 | | 0.14 | 0.47 | 0.49 | 0.93 | 0.34 | 2.75 | 0.10 | 1.63 | 0.20 | 0.88 | 0.35 |
| ELYCAN | | 1 | 0.22 | | 0.64 | 0.30 | 0.59 | 1.76 | 0.19 | 0.78 | 0.38 | 0.09 | 0.77 | 0.02 | 0.90 |
| BOUGRA | | 1 | 5.17 | | **0.02** | 0.09 | 0.77 | 1.99 | 0.16 | 0.59 | 0.44 | 0.09 | 0.76 | 6.85 | **0.01** |
| PANVIR | | 1 | 0.09 | | 0.76 | 3.11 | 0.08 | 0.02 | 0.89 | 7.88 | **0.01** | 4.92 | **0.03** | 0.79 | 0.38 |
| AMOCAN | | 1 | 0.06 | | 0.81 | 2.67 | 0.10 | 0.23 | 0.63 | 0.03 | 0.86 | 0.84 | 0.36 | 1.02 | 0.31 |
| DALCAN | | 1 | <0.01 | | 0.99 | 1.76 | 0.19 | 0.01 | 0.92 | 1.40 | 0.24 | 3.00 | **0.08** | 0.04 | 0.85 |
| DALPUR | | 1 | 0.20 | | 0.66 | 0.02 | 0.89 | 1.58 | 0.21 | 0.79 | 0.37 | 5.11 | **0.02** | 2.88 | **0.09** |
| DESILL | | 1 | 2.09 | | 0.15 | 0.23 | 0.63 | 0.01 | 0.92 | 3.17 | 0.08 | 0.48 | 0.49 | 0.16 | 0.69 |
| DESCAN | | 1 | <0.01 | | 0.99 | 1.48 | 0.23 | 2.02 | 0.16 | <0.01 | 0.98 | 0.11 | 0.74 | 0.94 | 0.33 |
| CHAFAS | | 1 | 0.03 | | 0.86 | 1.52 | 0.22 | 1.14 | 0.29 | 0.02 | 0.89 | <0.01 | 0.98 | 0.85 | 0.36 |
| LIAPYC | | 1 | 0.15 | | 0.70 | 0.26 | 0.61 | 0.14 | 0.71 | 2.34 | 0.13 | 2.22 | 0.14 | 0.12 | 0.73 |
| CORTIN | | 1 | 2.89 | | 0.09 | 0.54 | 0.46 | 1.94 | 0.17 | 0.27 | 0.60 | 0.08 | 0.78 | 1.80 | 0.18 |
| ECHPAL | | 1 | 1.65 | | 0.20 | 1.41 | 0.24 | 1.05 | 0.31 | 0.06 | 0.80 | 0.05 | 0.82 | 0.45 | 0.50 |
| EUPALT | | 1 | 0.30 | | 0.58 | 0.14 | 0.70 | 0.21 | 0.65 | 0.11 | 0.74 | 0.03 | 0.85 | 0.32 | 0.57 |
| SILINT | | 1 | 2.22 | | 0.14 | 0.18 | 0.67 | 3.08 | **0.08** | 0.30 | 0.59 | 0.73 | 0.39 | 2.03 | 0.16 |
| HELMOL | | 1 | 0.24 | | 0.63 | 3.38 | 0.07 | 1.42 | 0.24 | 0.04 | 0.85 | 0.78 | 0.38 | 0.06 | 0.81 |
| **Phyl PlantFam*SpRich** | | 3 | 0.10 | | 0.96 | 2.16 | 0.09 | 0.56 | 0.64 | 1.02 | 0.38 | 1.21 | 0.31 | 0.79 | 0.50 |
| **PlantFam *Precip** | | 3 | 1.19 | | 0.31 | 0.28 | 0.84 | 4.16 | **0.01** | 0.30 | 0.82 | 0.20 | 0.90 | 0.48 | 0.70 |
| **SpRich* Precip** | | 1 | 0.04 | | 0.85 | 0.07 | 0.79 | 0.64 | 0.42 | 0.20 | 0.65 | 0.02 | 0.89 | 2.58 | 0.11 |
| **PlantFam *SpRich* Precip** | | 3 | 0.33 | | 0.81 | 1.52 | 0.21 | 2.27 | 0.08 | 1.54 | 0.21 | 3.05 | **0.03** | 0.09 | 0.97 |
| Residuals | | 202 |  | |  |  |  |  |  |  |  |  |  |  |  |

Table S3- **Relative abundance of functional guilds.** Generalized linear model response of relative abundance for fungal saprotrophs, pathogens, and rhizobial bacteria to the full planting model.

| Relative abundance | | Saprotrophs:Fungi | | Pathogens:Fungi | | Rhizobia:Bacteria | |
| --- | --- | --- | --- | --- | --- | --- | --- |
|  | **Df** | **F value** | **Pr(>F)** | **F value** | **Pr(>F)** | **F value** | **Pr(>F)** |
| Block | 1 | 8.75 | <0.01 | 0.23 | 0.63 | 128.13 | <0.01 |
| **PlantFam** | 3 | 0.54 | 0.66 | 2.20 | **0.09** | 4.57 | **<0.01** |
| **SpRich** | 1 | 0.65 | 0.42 | 0.05 | 0.83 | 0.41 | 0.52 |
| **Precip** | 1 | 5.85 | **0.02** | 1.03 | 0.31 | 1.21 | 0.27 |
| ANDGER | 1 | 0.39 | 0.53 | 2.49 | 0.12 | 0.70 | 0.40 |
| ELYCAN | 1 | <0.01 | 0.96 | 0.03 | 0.85 | 5.19 | **0.02** |
| BOUGRA | 1 | 2.18 | 0.14 | 0.06 | 0.80 | 2.52 | 0.11 |
| PANVIR | 1 | 4.75 | **0.03** | 3.26 | **0.07** | <0.01 | 0.96 |
| AMOCAN | 1 | 1.16 | 0.28 | 1.28 | 0.26 | 0.09 | 0.77 |
| DALCAN | 1 | 3.74 | 0.05 | 0.92 | 0.34 | <0.01 | 0.99 |
| DALPUR | 1 | 1.98 | 0.16 | <0.01 | 0.98 | 2.89 | 0.09 |
| DESILL | 1 | 0.01 | 0.92 | 0.21 | 0.65 | 1.57 | 0.21 |
| DESCAN | 1 | 2.50 | 0.12 | 0.14 | 0.71 | 0.48 | 0.49 |
| CHAFAS | 1 | 1.19 | 0.28 | 0.17 | 0.68 | 0.08 | 0.77 |
| LIAPYC | 1 | 0.49 | 0.48 | 0.04 | 0.83 | 1.54 | 0.22 |
| CORTIN | 1 | 0.07 | 0.80 | 0.01 | 0.91 | 0.06 | 0.81 |
| ECHPAL | 1 | 5.29 | 0.02 | 0.01 | 0.92 | 1.14 | 0.29 |
| EUPALT | 1 | 0.12 | 0.73 | 0.02 | 0.89 | 0.08 | 0.78 |
| SILINT | 1 | 1.11 | 0.29 | 0.03 | 0.87 | 0.01 | 0.93 |
| HELMOL | 1 | 1.49 | 0.22 | 2.85 | **0.09** | 0.04 | 0.84 |
| **PlantFam*SpRich** | 3 | 0.74 | 0.53 | 0.26 | 0.85 | 0.27 | 0.85 |
| **PlantFam *Precip** | 3 | 1.69 | 0.17 | 4.94 | **<0.01** | 0.64 | 0.59 |
| **SpRich* Precip** | 1 | 0.27 | 0.61 | 0.82 | 0.37 | 1.04 | 0.31 |
| **PlantFam *SpRich* Precip** | 3 | 0.11 | 0.96 | 0.85 | 0.47 | 1.78 | 0.15 |
| Residuals | 202 |  |  |  |  |  |  |

Table S4 – **Microbial component community response to experimental design.** Aitchison distance compositional response to the planting design for all microbial components.

| Composition (Aitchison) | | |  |  |  |  |  |  |  |  |  |  |
| --- | --- | --- | --- | --- | --- | --- | --- | --- | --- | --- | --- | --- |
|  | Fungal saprotrophs | | Fungal pathogens | | Oomycetes | | *Rhizobia* bact. | | Bacteria | | AM fungi | |
|  | **R2** | **Pr(>F)** | **R2** | **Pr(>F)** | **R2** | **Pr(>F)** | **R2** | **Pr(>F)** | **R2** | **Pr(>F)** | **R2** | **Pr(>F)** |
| Block | 0.01 | <0.01 | 0.02 | <0.01 | 0.01 | <0.01 | 0.02 | <0.01 | 0.03 | <0.01 | 0.02 | <0.01 |
| **PlantFam** | 0.02 | **<0.01** | 0.01 | 0.85 | 0.01 | 0.66 | 0.01 | 0.60 | 0.01 | **0.06** | 0.02 | **<0.01** |
| **SpRich** | 0.01 | **0.02** | <0.01 | 0.53 | <0.01 | 0.26 | 0.01 | 0.09 | <0.01 | 0.78 | <0.01 | 0.58 |
| **Precip** | 0.01 | **<0.01** | 0.01 | **<0.01** | 0.01 | **<0.01** | 0.01 | **<0.01** | 0.01 | **<0.01** | 0.02 | **<0.01** |
| ANDGER | <0.01 | 0.64 | <0.01 | 0.25 | <0.01 | 0.32 | <0.01 | 0.39 | <0.01 | 0.10 | <0.01 | 0.73 |
| ELYCAN | 0.01 | **<0.01** | <0.01 | 0.31 | 0.01 | 0.13 | <0.01 | 0.70 | <0.01 | 0.55 | <0.01 | 0.06 |
| BOUGRA | 0.01 | 0.06 | 0.01 | 0.01 | <0.01 | 0.32 | 0.01 | 0.10 | 0.01 | **0.01** | <0.01 | 0.46 |
| PANVIR | <0.01 | 0.45 | <0.01 | 0.81 | 0.01 | **0.02** | 0.01 | **0.01** | <0.01 | 0.10 | <0.01 | 0.07 |
| AMOCAN | <0.01 | 0.52 | <0.01 | 0.73 | <0.01 | 0.87 | <0.01 | 0.47 | <0.01 | 0.53 | <0.01 | 0.22 |
| DALCAN | <0.01 | 0.15 | <0.01 | 0.61 | <0.01 | 0.76 | <0.01 | 0.55 | <0.01 | 0.10 | <0.01 | **0.03** |
| DALPUR | <0.01 | 0.41 | 0.01 | 0.20 | 0.01 | 0.23 | <0.01 | 0.80 | <0.01 | 0.28 | <0.01 | 0.49 |
| DESILL | 0.01 | **<0.01** | 0.01 | **<0.01** | <0.01 | 0.99 | <0.01 | 0.74 | <0.01 | 0.56 | 0.01 | **0.01** |
| DESCAN | 0.01 | **0.01** | <0.01 | 0.45 | <0.01 | 0.61 | <0.01 | 0.83 | <0.01 | 0.70 | <0.01 | 0.05 |
| CHAFAS | 0.01 | **0.05** | <0.01 | 0.75 | <0.01 | 0.81 | <0.01 | 0.89 | <0.01 | 0.54 | <0.01 | 0.26 |
| LIAPYC | <0.01 | 0.14 | 0.01 | **0.01** | 0.01 | **<0.01** | 0.01 | 0.09 | <0.01 | 0.10 | <0.01 | **0.06** |
| CORTIN | <0.01 | 0.48 | 0.01 | 0.15 | 0.01 | **0.08** | 0.01 | 0.07 | <0.01 | 0.33 | <0.01 | **0.04** |
| ECHPAL | <0.01 | 0.10 | <0.01 | 0.28 | <0.01 | 0.99 | <0.01 | 0.65 | <0.01 | 0.65 | <0.01 | 0.90 |
| EUPALT | <0.01 | 0.90 | <0.01 | 0.40 | <0.01 | 0.94 | <0.01 | 0.67 | <0.01 | 0.91 | <0.01 | 0.33 |
| SILINT | <0.01 | 0.43 | <0.01 | 0.67 | <0.01 | 0.54 | <0.01 | 0.60 | <0.01 | 0.11 | <0.01 | 0.18 |
| HELMOL | <0.01 | 0.62 | <0.01 | 0.94 | 0.01 | 0.10 | <0.01 | 0.19 | <0.01 | 0.34 | <0.01 | 0.11 |
| **PlantFam *SpRich** | 0.01 | **0.03** | 0.01 | 0.17 | 0.01 | 0.93 | 0.01 | 0.20 | 0.01 | 0.27 | 0.01 | 0.71 |
| **PlantFam *Precip** | 0.01 | 0.28 | 0.01 | 0.48 | 0.02 | 0.07 | 0.01 | 0.73 | 0.01 | 0.98 | 0.01 | 0.63 |
| **SpRich* Precip** | <0.01 | 0.93 | <0.01 | 0.79 | <0.01 | 0.27 | <0.01 | 0.24 | <0.01 | 0.83 | <0.01 | 0.84 |
| **PlantFam *SpRich* Precip** | 0.01 | 0.97 | 0.01 | 1.00 | 0.01 | 0.33 | 0.01 | 0.27 | 0.01 | 0.23 | 0.01 | 0.98 |
| Residuals | 0.83 |  | 0.84 |  | 0.84 |  | 0.84 |  | 0.83 |  | 0.83 |  |
|  | 1.00 |  | 1.00 |  |  |  | 1.00 |  | 1.00 |  | 1.00 |  |

Table S5 – Pearson’s product-moment correlation for composition permanova and diversity anova responses to planting design, compared to realized cover data. Correlation between variation explained due to each variable in the model were found to be highly correlated.


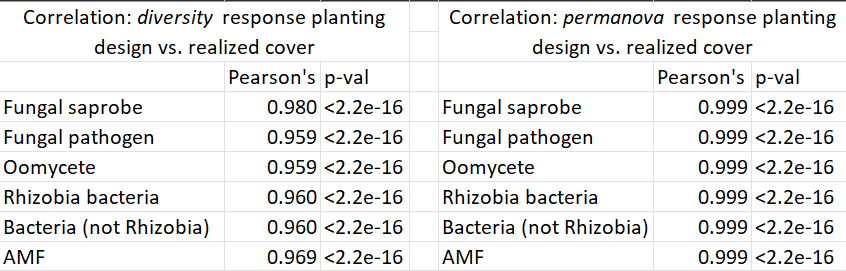


Table S6 – Beta-dispersion anova output for each community, shown for microbial communities that had significant differences to plant family composition (“PhyloFam”) or precipitation in the permanova.


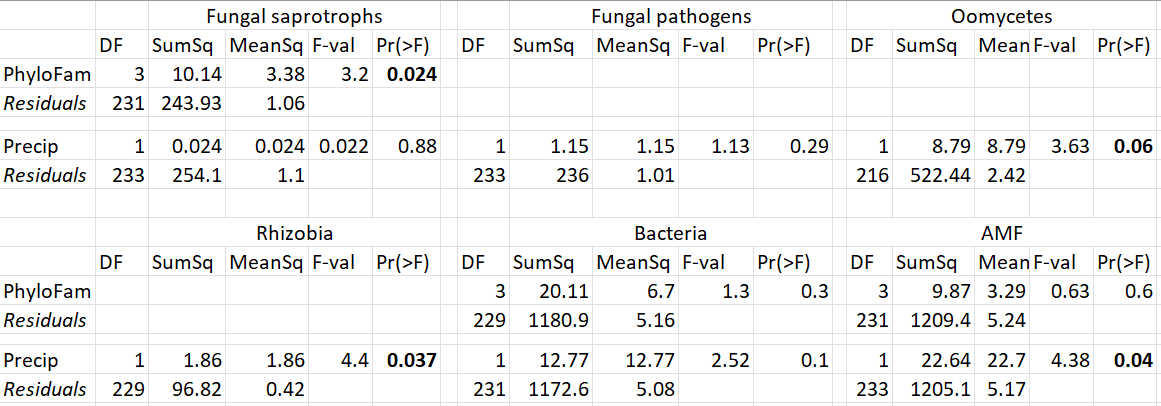


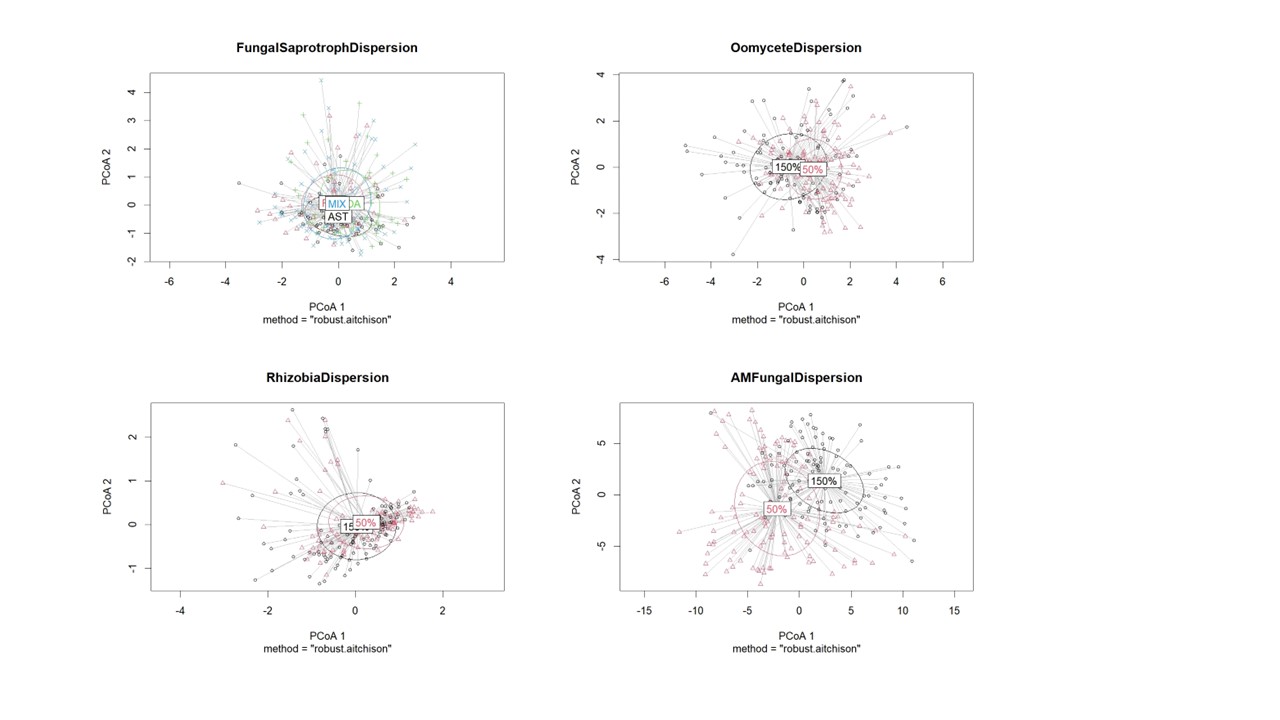


Figure S2 – Beta dispersion plots for significantly different spread in composition across fungal saprotrophs (top left), oomycetes (top right), rhizobia (bottom left) and AM fungi (bottom right).


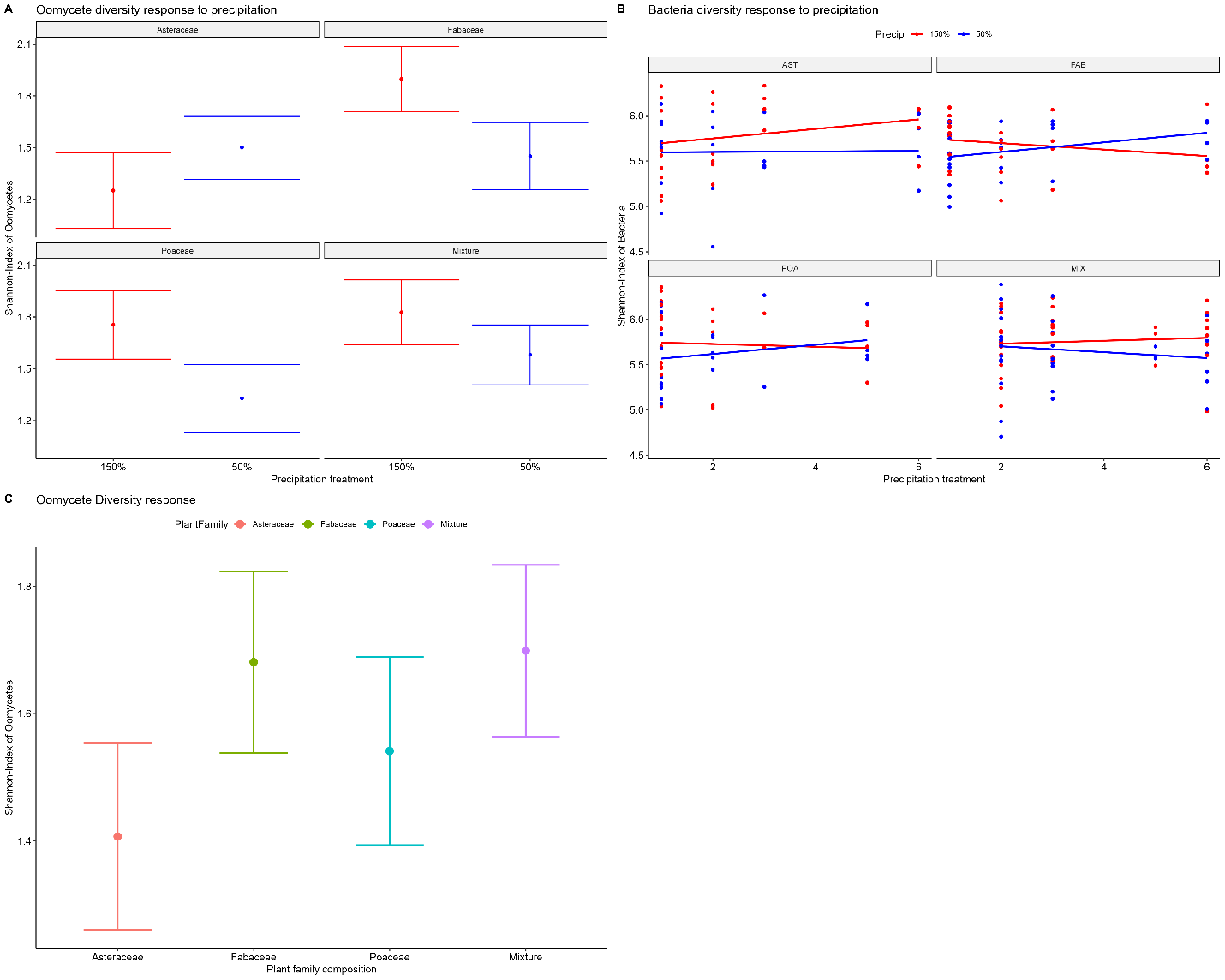


Figure S3 – Oomycete diversity (A) response to plant family composition and precipitation (Table 1, p=0.007). Bacteria diversity (B) response to interaction of plant family composition, precipitation, and plant species richness.


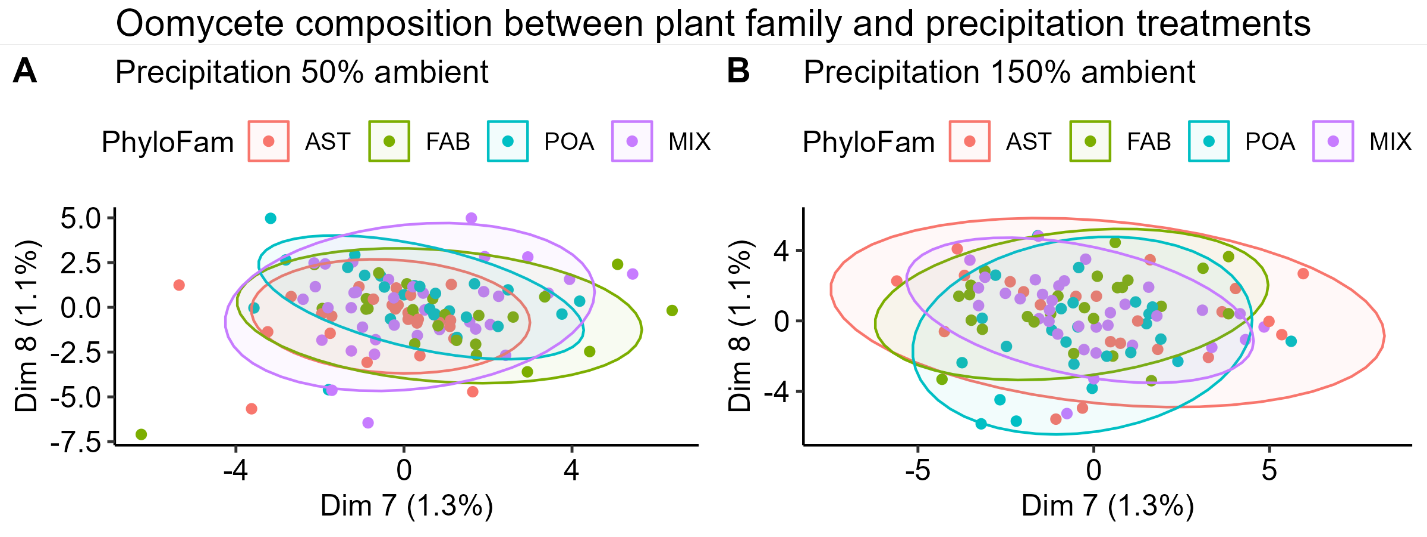


Figure S4 – Oomycete composition (Aitchison) response to interaction of plant family composition and precipitation treatments (Table 2, p=0.07). Plotted using dimensions that show divergence in response to interaction, 50% ambient precipitation (A) and 150% ambient (B).
